# Dibromopinocembrin and Dibromopinostrobin Are Potential Anti-Dengue Leads with Mild Animal Toxicity

**DOI:** 10.3390/molecules25184154

**Published:** 2020-09-11

**Authors:** Siwaporn Boonyasuppayakorn, Thanaphon Saelee, Peerapat Visitchanakun, Asada Leelahavanichkul, Kowit Hengphasatporn, Yasuteru Shigeta, Thao Nguyen Thanh Huynh, Justin Jang Hann Chu, Thanyada Rungrotmongkol, Warinthorn Chavasiri

**Affiliations:** 1 Applied Medical Virology Research Unit, Department of Microbiology, Faculty of Medicine, Chulalongkorn University, Bangkok 10330, Thailand; thanaphon.saelee@gmail.com; 2 Interdisciplinary Program in Microbiology, Graduate School, Chulalongkorn University, Bangkok 10330, Thailand; peerapat.visitchanakun@gmail.com; 3 Translational Research in Inflammation and Immunology Research Unit, Department of Microbiology, Faculty of Medicine, Chulalongkorn University, Bangkok 10330, Thailand; aleelahavanit@gmail.com; 4 Center for Computational Sciences, University of Tsukuba, 1-1-1 Tennodai, Tsukuba, Ibaraki 305-8577, Japan; heng.kowit@gmail.com (K.H.); shigeta@ccs.tsukuba.ac.jp (Y.S.); 5 Center of Excellence in Natural Products Chemistry, Department of Chemistry, Faculty of Science, Chulalongkorn University, Bangkok 10330, Thailand; thao.huynhthanh94@gmail.com (T.N.T.H.); warinthorn.c@chula.ac.th (W.C.); 6 Laboratory of Molecular RNA Virology and Antiviral Strategies, Department of Microbiology and Immunology, Yong Loo Lin School of Medicine, National University of Singapore, Singapore 117545, Singapore; miccjh@nus.edu.sg; 7 Program in Bioinformatics and Computational Biology, Graduate School, Chulalongkorn University, Bangkok 10330, Thailand; thanyada.r@chula.ac.th; 8 Biocatalyst and Environmental Biotechnology Research Unit, Department of Biochemistry, Faculty of Science, Chulalongkorn University, Bangkok 10330, Thailand

**Keywords:** dengue virus, flavanone, flavonoid, antiviral drug, dengue methyltransferase, drug discovery, pinocembrin, pinostrobin

## Abstract

Dengue infection is one of the most deleterious public health concerns for two-billion world population being at risk. Plasma leakage, hemorrhage, and shock in severe cases were caused by immunological derangement from secondary heterotypic infection. Flavanone, commonly found in medicinal plants, previously showed potential as anti-dengue inhibitors for its direct antiviral effects and suppressing the pro-inflammatory cytokine from dengue immunopathogenesis. Here, we chemically modified flavanones, pinocembrin and pinostrobin, by halogenation and characterized them as potential dengue 2 inhibitors and performed toxicity tests in human-derived cells and in vivo animal model. Dibromopinocembrin and dibromopinostrobin inhibited dengue serotype 2 at the EC_50_s of 2.0640 ± 0.7537 and 5.8567 ± 0.5074 µM with at the CC_50_s of 67.2082 ± 0.9731 and >100 µM, respectively. Both of the compounds also showed minimal toxicity against adult C57BL/6 mice assessed by ALT and Cr levels in day one, three, and eight post-intravenous administration. Computational studies suggested the potential target be likely the NS5 methyltransferase at SAM-binding pocket. Taken together, these two brominated flavanones are potential leads for further drug discovery investigation.

## 1. Introduction

Mosquito-borne viruses, especially the four serotypes of dengue viruses, are health burden worldwide with 4.2 million case reports in 2019 [1]. Moreover, reported deaths increase from 960 to 4032 during 2000–15 [1]. Severe manifestations of dengue, such as plasma leakage, hemorrhage, multiple organ failure, and hypovolemic shock, are caused by a secondary heterotypic infection that leads to misled immunological response towards the previous serotype [2]. Moreover, evidence suggested that the viral load is higher in severe cases [3] because of increasing viral replication from partially neutralized antibody-mediated opsonization into a macrophage. Therefore, inhibiting viral replication could potentially alleviate the clinical severity. However, no specific drug is currently available for the treatment of severe dengue. 

The flaviviral replication starts with a specific binding between viral envelope (E) protein and cellular receptors [4]. This virus entered the cells via clathrin-mediated endocytosis [5] and escaped by fusion under acidic environment [6]. The positive-sense RNA genome also serves as an mRNA for translation *en bloc* to viral proteins utilized in subsequent viral replication, the termination of host protein translation, and suppression of interferon signaling. The virus particle is assembled in the endoplasmic reticulum [7] and travels through Golgi complex for maturation by furin cleavage. The viral particle is released by budding out of the infected cells [8]. Potential drugs were designed and developed to interfere with those steps of viral replication or to target the pathological activation of pro-inflammatory cytokines. 

Flavonoids were plant-derived secondary metabolites with various biological activities, including antivirals and anti-inflammation. A previous report suggested that halogenation of chrysin (5,7-dihydroxyflavone) enhances the efficacy against dengue and Zika viruses [9] in the cell-based system. However, two halogenated chrysins exhibited limited aqueous solubility at the administrative dose, preventing further animal toxicity investigation. To overcome the challenge, a flavanone substrate with higher solubility was considered as a replacement. Moreover, pinocembrin (5,7-dihydroxyflavanone), was previously reported as a potential inhibitor of dengue [10,11] and Zika viruses [12] with possible targets at viral NS2B-3 protease [12,13,14]. Moreover, anti-flaviviral inhibition was also reported in other flavanones, such as pinostrobin, naringenin, hesperitin, and their derivatives. In this study, we hypothesized whether halogenation of selected potential flavanones, pinocembrin and pinostrobin, would enhance antiviral efficiency similar to those of the flavones, and increase aqueous solubility to be eligible for toxicity studies in cell-based and animal model. 

## 2. Results

### 2.1. Compound Syntheses and Identification 

#### 2.1.1. Synthetic Protocol

**Figure 1:**
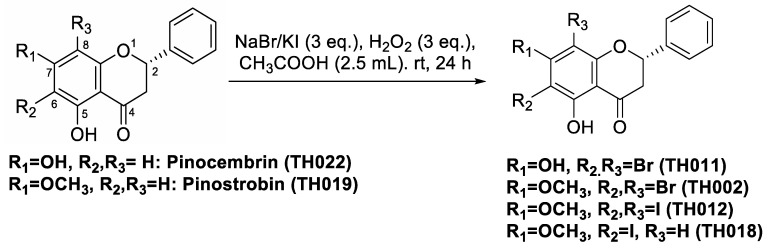
Chemical structures of pinocembrins; **TH011** and **TH022**, and pinostrobins; **TH002**, **TH012**, **TH018**, and **TH019**, and the halogenation scheme.

The bromination and iodination of pinostrobin (**TH019**) and pinocembrin (**TH022**) were adapted from the protocol previously described [15] (Figure 1). Following the reactions, 6,8-dibromopinostrobin (**TH002**), 6,8-dibromopinocembrin (**TH011**), 6,8-diiodopinocembrin (**TH012**), and 6-iodopinostrobin (**TH018**) were isolated using silica gel chromatography, yielding 96%, 93%, 73%, and 10%, respectively.

#### 2.1.2. Identification by NMR

The two halogen atoms of **TH011**, **TH002**, and **TH012** were identified by the loss of two proton signals at 6-, and 8-positions at δ_H_ 6.07 and 5.94 ppm of pinocembrin (**TH022**) and pinostrobin (**TH019**), respectively (Supplementary Figures S1–S3). Furthermore, an addition of two halide atoms created a large shift of chelated hydroxyl proton at position 5 identified as a characteristic signal of the spectra. Similarly, 6-iodopinostrobin (**TH018**) (Supplementary Figure S4) was recognized by the loss of one proton signal at 6-position and shared the similar characteristic signal of a hydroxyl proton shift at position 5. 

### 2.2. Efficacies and Cytotoxicity of Pinocembrin and Pinostrobin Derivatives

Halogenated pinocembrin and pinostrobin were tested against dengue virus serotype 2 (DENV2), New Guinea C strain in LLC/MK2 cells (Table 1). Cytotoxicities of the compounds were also assessed (Table 1 and Table 2). The selectivity index (SI) is a ratio of CC_50_ and EC_50_ indicating the safety range of the particular compound to effectively inhibit the virus replication without being cytotoxic to the particular cell line. The results showed that all halogenated derivatives were more potent than their original compounds. The most potent compound was 6,8-dibromopinocembrin (**TH011**) with an effective concentration (EC_50_) of 2.0640 ± 0.7537 µM and a SI of 32.5621. Additionally, **TH011** consistently inhibited another DENV2 strain, 16681, in Huh-7 cells at an EC_50_ of 4.5936 ± 1.8520 µM and a SI of 10.5201. The Huh-7 CC_50_ was 48.3277 ± 4.1129 µM (Table 2). Besides LLC/MK2 and Huh-7, **TH011** displayed mild cytotoxicity to THP-1, HepG2, and HEK-293 cell lines (Table 2). The original pinocembrin (**TH022**) was previously reported as DENV2, and Zika viruses inhibitor with moderate efficacies [16] at the EC_50_s of 15.45, and 17.4 µM, respectively [11,17]. Therefore, we concluded that the dibromopinocembrin (**TH011**) was more potent than its original compound for DENV2 inhibition.

Brominated (**TH002**) and iodinated (**TH012** and **TH018**) pinostrobins similarly inhibited the DENV2 NGC infectivity at the EC_50_ around 2.8–5.8 µM (Table 1) exceeding their original compound. However, both iodopinostrobins (**TH012** and **TH018**) were partially dissolved when the concentration exceeded 10 and 25 µM, respectively, thus limiting their cytotoxic studies. Therefore, bromopinostrobin (**TH002**) was the most promising candidate, with the SI of 17.0745 (Table 1). **TH002** was further analyzed using an alternative system of DENV2 16681-infected Huh-7 cells. The EC_50_ and SI results were 3.1933 ± 1.0971 µM, and 23.9342, respectively, being relatively similar to those of DENV2 NGC- infected LLC/MK2 cells. Cytotoxicity against various cell lines (Table 2) revealed that **TH002** was more toxic to THP1 and HepG2, in contrast to those of **TH011**. From the efficacies and selectivity results, we concluded that brominated pinocembrin (**TH011**) and pinostrobin (**TH002**) were promising candidates for further in vivo toxicity study.

### 2.3. In Vivo Toxicity of Pinocembrin and Pinostrobin Derivatives

**Figure 2:**
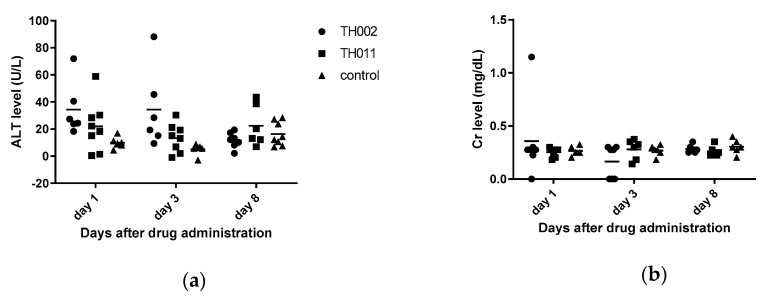
In vivo toxicity of adult C57BL/6: (**a**) Alanine aminotransferase level represented the hepatotoxicity; (**b**) Creatinine level represented the renal toxicity. The 10% DMSO in normal saline solution was a vehicle control.

Flavonoids generally have low bioavailability due to interactions with digestive enzymes and intestinal microorganisms [18]. Flavanones were metabolized to flavone by CYP2A6 and CYP2A13 in the liver [19] and mainly excreted in urine within 4–8 h [20]. In this study, the compounds were intravenously administered to avoid intestinal interactions; therefore, the toxicity would be observed in nonmetabolized forms of **TH011** and **TH002**. All of the compounds and control were prepared in normal saline solution and administered to C57BL/6 mice at 10 mg/kg, or around 100–200 times higher than the EC_50_s previously described. The vehicle control was 10% dimethylsulfoxide (DMSO) in normal saline solution. General appearance, plasma alanine aminotransferase (ALT), and plasma creatinine (Cr) representing the liver and kidney functions, respectively, were monitored on days one, three, and eight after administrations (Figure 2a,b). Overall, all of the animals were active without any signs of distress or abnormalities suggesting that they were well-tolerated to the drugs. However, ALT levels of the **TH002** group were mildly elevated on day 1 and 3, suggesting asymptomatic drug-induced hepatitis. The level was decreased to normal on day 8 suggesting a full recovery. In contrast, Cr levels did not change in any group, suggesting no detected renal toxicity. Noted that the compounds were challenged to the animals at around 100 times higher than the effective doses. 

### 2.4. Molecular Target Identification 

**Figure 3:**
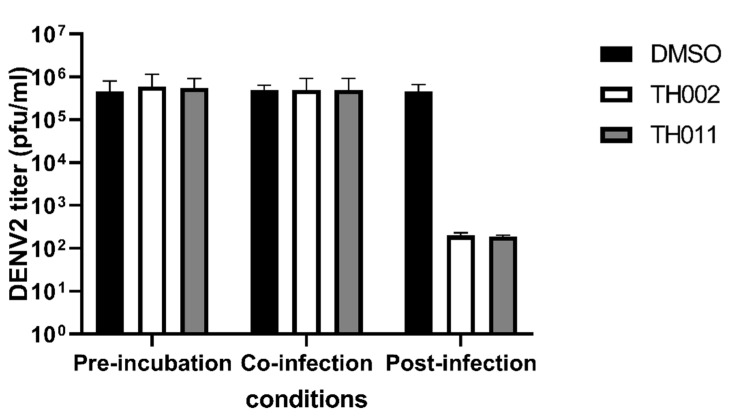
Attachment inhibition assay, DENV2 (16681)-infected Huh-7 cells were treated with 10 µM TH002, TH011, or dimethylsulfoxide (DMSO) before, during, or after infection. Three independent experiments were performed to verify the results.

Original pinocembrin (**TH022**) was previously characterized as a ZIKV post-entry inhibitor in a cell-based system [17], and pinostrobin (**TH019**) was a non-competitively inhibit DENV NS2B-3 protease (pro) activity in vitro [13]. Other flavanone derivatives were previously reported as inhibitors of the viral envelope (E) [11], NS2B-3 pro [14], NS5 polymerase (pol) [21], and cellular factors involving in viral replication [22]. In this section, the halogenated flavanones were characterized for potential molecular targets. Attachment inhibition showed the major targets of both compounds located at post-infection (Figure 3). The compounds did not inhibit DENV envelope-induced fusion under acidic pH (Supplementary Figure S5). Therefore, it was likely that **TH011** and **TH002** would inhibit the DENV translation and replication similar to the previously characterized pinocembrin (**TH022**) in ZIKV replicon [17]. 

**Figure 4:**
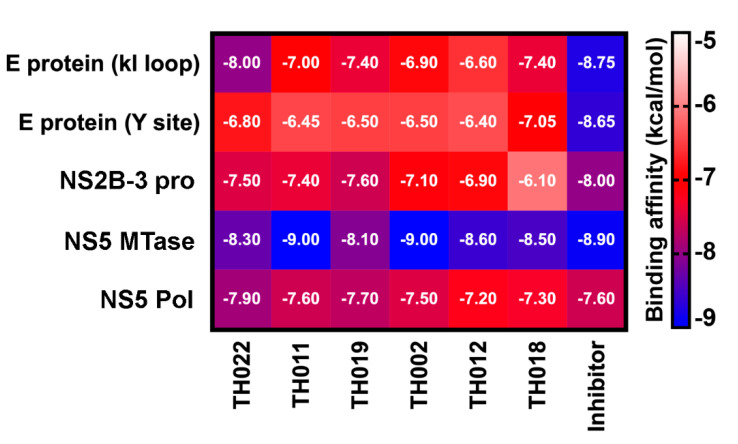
Binding affinity (kcal/mol) for the studied pinocembrins (**TH022** and **TH011**) and pinostrobins (**TH019, TH002, TH012,** and **TH018**) in comparison with the known inhibitors of DENV protein targets: E protein at kl loop and Y site (FN5Y [11]), NS2B-3 pro at allosteric site (compound 9 [25]), NS5 MTase at SAM binding site (Sinefungin [27]), and NS5 Pol at the active site (NITD-107 [26]), predicted by molecular docking method using AutoDock VinaXB.

A computational approach was executed to narrow down the possibility of potential targets for our focused compounds becasue hundreds of cellular proteins and interactions are involved in flaviviral replication. The binding affinity of halogenated and original flavanones on DENV2 E, NS2B-3 pro, NS5 methyltransferase (MTase), and NS5 RNA-dependent RNA polymerase (Pol) was predicted and compared with the known inhibitors while using AutoDock VinaXB software in which the halogen bonding parameters were taken into account [23]. The docking results of the four protein targets (Figure 4) showed that the two potent compounds, dibromopinocembrin (**TH011**) and dibromopinostrobin (**TH002**), preferentially interacted at the SAM-binding site of NS5 MTase, in which their binding energy of −9.0 kcal/mol slightly exceeded that of sinefungin that is commonly used to inhibit the DNA MTases. Although the pinostrobin and one of pinocembrin analogs were previously reported to be active against NS2B-3 pro and NS5 pol, respectively [21,24], none of the halogenated analogs in this study could bind to these targets stronger than their parent compounds **TH022** and **TH019**, as well as the compound 9 [25] and NITD-107 [26], which were protease and NS5 Pol inhibitors, respectively. Besides, the binding affinity of **TH011** and **TH002** at both kl loop and Y site of the DENV E protein were relatively lower than that of the potent flavanone FN5Y [11] in consistent with no fusion inhibition observed (Supplementary Figure S5). 

**Figure 5:**
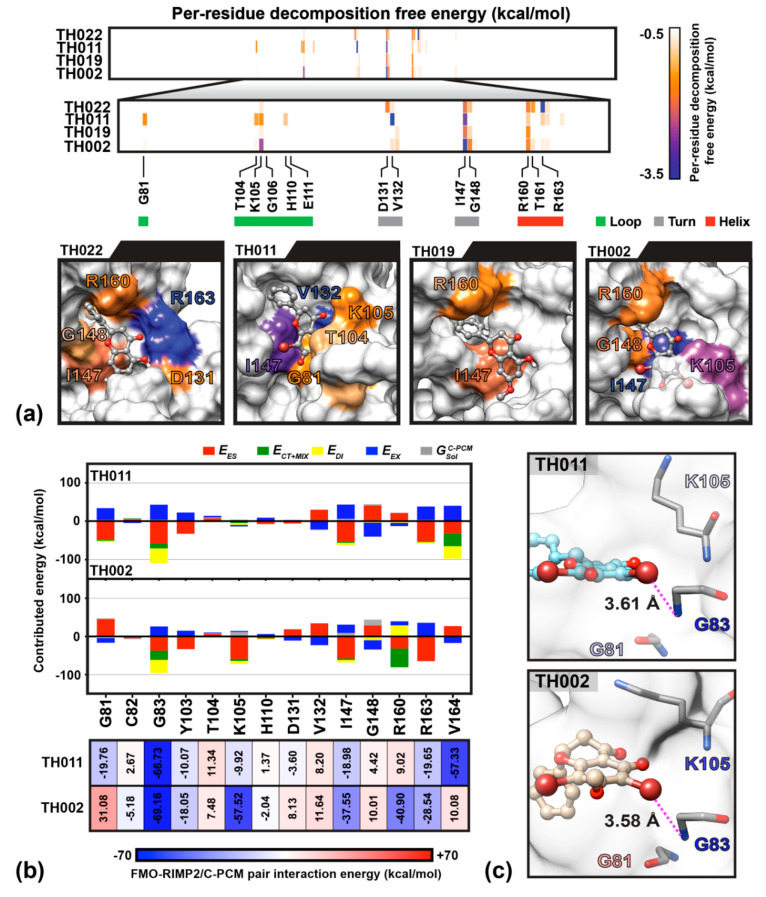
(**a**) The important residues and molecular conformation of the four flavanones binding NS5 methyltransferase (MTase) at the SAM pocket obtained from Molecular Mechanics-Generalized Born Surface Area method (MM-GBSA) per-residue decomposition free energy calculation on the MD trajectories during 200–300 ns. The surface color for all systems associated with energy is labeled from −3.5 to −0.5 kcal/mol (blue-orange-white). The residues with per-residues decomposition free energy lower than −1 kcal/mol are labeled. (**b**) FMO-RIMP2/C-PCM pair interaction energy (kcal/mol) of surrounding residues with the two potent dibrominated compounds and (**c**) averaged distance (Å) measured between the G83 backbone nitrogen and the Br^6^ atom of dibrominated flavanones; **TH011** and **TH002**.

The molecular dynamics study showed that the dibrominated flavanones; **TH011** and **TH002**, were consistently bound to the SAM-binding site of NS5 MTase throughout the 300-ns (data not shown). To evaluate the stability, binding pattern, and interaction profile of each compound, the per-residue decomposition and binding free energy (Δ$G_{bind}$) calculations were performed on the last 100-ns snapshots of ligand-MTase complexes using the Molecular Mechanics-Generalized Born Surface Area method (MM-GBSA) (Figure 5). As a result of the bromine substitutions on the chromone ring of flavanones, the binding affinities of **TH011** and **TH002** (Δ$G_{bind}$ of −12.50 ± 1.2 and −12.49 ± 1.2 kcal/mol) were significantly greater than their parent compounds pinocembrin and pinostrobin (−6.65 ± 1.6 and −0.58 ± 2.1 kcal/mol) (Figure 5a). The phenyl ring of the two dibrominated compounds was stabilized by the αD-helix in particular residues R160, T161, R163 and V164, while only the chromone ring of **TH011** orientated at almost the same position as the purine ring of sinefungin intensively interacted with the following residues; G81, T104, K105, H110, V132, and I147. The efficient computational technique based on the FMO-RIMP2/C-PCM method was then carried out to describe electrostatics, polarization, dispersion and charge transfer between the two potent dibrominated flavanones and NS5 MTase. Besides the 12 binding residues that were observed by MM-GBSA calculation, there were two additional key residues G83 and V164 (Figure 5b). At SAM binding site on MTase, the G83 showed the highest contribution to **TH011** (−66.73 kcal/mol) and **TH002** (−69.16 kcal/mol), followed by V164 in TH011 (−57.33 kcal/mol) and K105 in **TH002** (−57.52 kcal/mol). The essential interaction of TH011 with G83 backbone was from the electrostatic attraction ($E_{ES}$ −57.96 kcal/mol) and the halogen interaction contributed by dispersion ($E_{DI}$ −39.20 kcal/mol) and charge transfer ($E_{CT+MIX}$ −12.65 kcal/mol) to the Br^6^ on the chromone ring (averaged distance of 3.61 Å in Figure 5c). V164 attributed to the phenyl ring of TH011 by $E_{ES}$, $E_{DI}$ and $E_{CT+MIX}$ of ~−32 kcal/mol per interaction, while each of the three residues G81, I147 and R163 interplayed for almost −20 kcal/mol, mainly through $E_{ES}$. Changing from the hydroxyl group in **TH011** to the methoxy group in **TH002** on the C^7^ position between the two bromines on the chromone ring led to alternative binding interactions. For example, a decreased contribution in terms of electrostatic interaction and dispersion (−37.42 and −34.47 kcal/mol) in G83 was compensated by an enhanced charge transfer (−23.75 kcal/mol). Remarkable higher stabilization was also found at the residues K105 and R160 (~50 kcal/mol), I147 (~20 kcal/mol), and R163 (~10 kcal/mol), whereas the residues G81, D131, and V164 repulsed the **TH002** binding. Altogether, the finding suggested that the bromine substitution was essential for lead-compound optimization.

**Figure 6:**
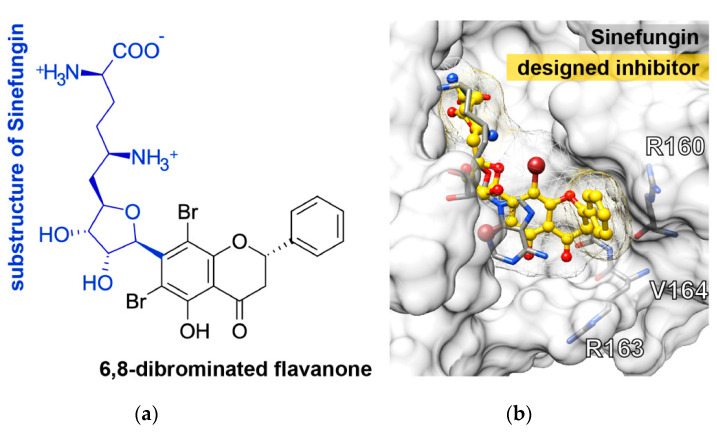
(**a**) Two-dimensional (2D) structure of the newly designed compound and (**b**) its docked conformation in the SAM binding region of dengue NS5 MTase domain compared to the crystal structure of sinefungin in complex with NS5 MTase.

From the obtained results, the 6,8-dibrominated flavanone should be kept as the core structure instead of the purine ring of sinefungin, while the chromone ring was optimized by replacing its tail, (2R,5R)-2,5-diamino-6-[(3S,4R)-3,4-dihydroxy-5-methyloxolan-2-yl] hexanoic acid, on the C^7^ atom (Figure 6a). From docking study, the newly designed dibrominated flavanone strongly interacted with the surrounding residues and occupied well in the SAM-binding pocket (Figure 6b) in a correspondence with higher binding affinity (−10.3 kcal/mol) than either sinefungin (−8.9 kcal/mol) or **TH011** (−9.0 kcal/mol). 

## 3. Discussion

A flavanone structure is a member of flavonoid (C6-C3-C6) with the presence of a chiral center at C2 and the absence of the C2–C3 double bond [28]. They are plant-derived secondary metabolites originated from chalcones in the flavonoid biosynthetic pathway, and still highly reactive for further structural modifications, such as dehydrogenation, glycosylation, hydroxylation, etc. They are key mediators of various biological processes in plants, such as nitrogen fixation, photosynthesis, energy transfer, and anti-oxidation [15]. Moreover, their activities extended towards anti-cancer, antivirals [10,12,17], and anti-inflammation [29,30]. Generally, flavanones are further metabolized to flavones by the addition of a double bond to C2–C3 of ring C, thus decreasing the aqueous solubility (Supplementary Figure S6) [31]. 

A previous study reported that halogenated chrysins (6,8-dibromo-, and 6,8-diiodo-5,7-dihydroxyflavone) were potential dengue and Zika inhibitors [9], but the animal toxicity could not be accessed, because of the insolubility issue (data not shown). Halogen atoms have a high electronegativity (EN) property to stably bind to the residues and were hardly metabolized by catalytic enzymes. The question was raised as to whether the halogenated flavanones would also enhance the flaviviral inhibition like halogenated flavones, with an advantage of increasing aqueous solubility. Pinocembrin and pinostrobin were chosen to halogenation for their structural similarities to chrysin. The synthesis was conducted in a one-step process and both bromoflavanones yielded > 90% by silica gel chromatography. Generally, bromoflavanones (**TH002** and **TH011**) were completely dissolved in culture media, DMSO, and NSS, in contrast, iodoflavanones were re-crystalized after 2–3 days incubation in the cell culture media (data not shown). Despite the solubility issue, all of the halogenated derivatives were similarly effective to inhibit DENV2 NGC at 2.06–5.83 µM (Table 1). Moreover, both **TH011** and **TH002** were also effectively inhibited DENV2 16,681 at 4.59 and 3.19 µM, respectively, in a different cell line system. The consistent results suggested that the bromination indeed potentiated the inhibitory effect of the flavanones. (Table 1) [11]. Taken together, the dibromopinocembrin (**TH011**) and dibromopinostrobin (**TH002**) were selected for further cell-based and in vivo toxicity analysis. 

**TH002** and **TH011** were both tested for cytotoxicities in LLC/MK2, THP-1, HEK, HepG2, and Huh-7 cell lines representing DENV common targets (Table 1 and Table 2). Noted that LLC/MK2 and HEK cell lines were originated from renal epithelium of *Macaca mulatta* (rhesus monkey) and human embryo, respectively; whereas HepG2 and Huh-7 cell lines were originated from human hepatocellular carcinoma. Despite various performance, the cytotoxicities to all cell lines were > 50 µM, or at least 10 times higher than the efficacies of particular compounds (2.06–5.83 µM). The original pinocembrin reduced endoplasmic reticulum stress and apoptosis by suppressing C/EBP homologous protein (CHOP), and caspase-3 expression/activity, respectively [32]. It is possible that the pinocembrin would interfere with DENV-induced ER stress, thereby reducing viral replication. Other positive-sense RNA viruses those replication involving ER-stress induction, such as Zika, Chikungunya, and enteroviruses, were also inhibited by pinocembrin [17]. Moreover, pinocembrin and pinostrobin were well known to downregulate pro-inflammatory cytokines (e.g., TNF-α, IFN-γ, IL-6) in monocyte/macrophage, thus alleviated the burden of dengue immunopathogenesis. Likely, the halogenated derivatives would also target similar cellular pathways as their parent compounds. 

Moreover, in vivo toxicity showed that **TH002**-induced hepatotoxicity was observed by ALT elevation on day 1 and 3. The liver enzyme returned to normal level on day 8 (Figure 2a) suggesting a self-limited and total recovery of the liver. The elevated ALT was not obviously seen in **TH011** and DMSO control group. Note that both of the compounds were intravenously administered at 10 mg/kg, or about 100 times of the EC_50_s. Therefore, the administered dose was intended to exceed a therapeutic level in blood circulation. After intravenous administration, pinocembrin was rapidly cleared from the circulation with a half-life of 12–14 min. in the rat (10 mg, single dose) and 47 min. in human (20 mg, iv drip) [32], mainly by CYP2A6 and CYP2A13 metabolism [19] and renal clearance. Therefore, further in vivo efficacy should include the multiple doses of administration in order to maintain the therapeutic level during viremia or febrile phase. Moreover, glycosidation of flavanone [33] and flavone [34] potentiated the antiviral efficacies resulting from increasing bioavailability and intestinal absorption [33,35]. The addition of carbohydrate moiety to the compounds could also be an option for further structural modification.

Our previous finding was dibromo- and diiodochrysins (5,7-dihydroxyflavones) potentiated the DENV1-4 inhibition, as well as a broad-spectrum inhibition to Zika viruses [9]. In this work, the halogenated flavanones similarly potentiated the DENV2 inhibition (NGC and 16681) in two cell line systems (Table 1 and Table 2). Although the definitive molecular targets are still unknown, the compounds mainly inhibited at post-infection (Figure 3), similar to the parent compound, pinocembrin, and Zika virus at 17.4 µM [17]. Another report suggested that pinocembrin inhibited at DENV NS3 protease activity (IC_50_ 286.90 µg/mL) [14], but not inhibit DENV NS5 polymerase activity [21]. Similarly, pinostrobin also inhibited DENV NS3 at the IC_50_ of 90.48 µg/mL) [14], or the *Ki* of 345 ± 70 µM [24]). Other flavanones, such as 6-methylpinostrobin inhibited DENV2 and 4 (EC_50s_ 15.99 ± 5.38 and 11.70 ± 6.04 µM, respectively), by interfering with conformational change of DENV envelope proteins during fusion [11]. Therefore, we included dengue envelope (E), protease (NS2B-3 pro), methyltransferase (NS5 MTase), and RNA-dependent RNA polymerase (NS5-pol) to molecular docking panel (Figure 4). Results showed that the most likely target of **TH011** and **TH002** was NS5 MTase for the strongest binding affinities (−9.0 kcal/mol) as compared to the parent compounds (−8.3 and −8.1 kcal/mol) and the inhibitor, sinefungin (−8.9 kcal/mol). The second possible target was NS5-pol, whereas E and NS2B-3 pro were unlikely for their moderate affinity contribution. The results were then confirmed by MD simulation (Figure 5). G83 was a key residue interacting with the Br^6^ atom of dibrominated flavanones. Note that the viral MTase activities were critical to the replication of positive-sense RNA viruses in the cytoplasmic compartment, whereby the host MTases were unavailable. Additionally, the eukaryotic ribosomal system is tightly stringent over the methylated capped species (cap 1, N7 and 2′-O methylation) for mRNA translation. Therefore, the in vitro N7 and 2′-O NS5 methyltransferase assays mimicking both of methylation activities could be used for further investigation [36]. 

## 4. Materials and Methods 

### 4.1. Compound Synthesis

#### 4.1.1. General Procedure for Bromination

A 30% aqueous solution of H_2_O_2_ (3.0 eq.) was added to the flask of flavanone (0.1 mmol) and NaBr (3.0 eq.) in CH_3_COOH (2.5 mL); then, the mixture was stirred at room temperature for 24 h. The reaction was monitored by TLC. At the endpoint, the crude was treated with Na_2_S_2_O_3_ and extracted with EtOAc (3 × 10 mL). The reunited organic fractions were dried over anhydrous Na_2_SO_4_; after filtration, the solvent was evaporated under reduced pressure. Final products were isolated and then purified by chromatographic column while using hexane: EtOAc: acetone = 60:40:4 as eluent.

#### 4.1.2. General Procedure for Iodination 

The starting materials were isolated from Boesenbergia rotunda (L.) Mansf. (Zingiberaceae). They are enantiomerically pure by nature. A 30% aqueous solution of H_2_O_2_ (3.0 equiv.) was added to the flask of flavanone (0.1 mmol) and KI (3.0 equiv.) in CH_3_COOH (2.5 mL); then, the mixture was stirred at room temperature for 24 h. The reaction was monitored by TLC. At the end, the crude was treated with Na_2_S_2_O_3_ and extracted with EtOAc (3 × 10 mL). The separated organic fractions were dried over anhydrous Na_2_SO_4_; after filtration, the solvent was evaporated under reduced pressure. The final products were purified by chromatographic column using hexane: EtOAc: acetone = 60:40:4 as eluent.

#### 4.1.3. Identification by Mass Spectrometry, ^1^H and ^13^C-NMR 

All of the NMR spectra (^1^H and ^13^C-NMR) were recorded in deuterated chloroform (CDCl_3_), acetone-*d*_6_, methanol-*d*_4_ (MeOD) or dimethylsulfoxide-*d*_6_ (DMSO-*d*_6_) on a Bruker AV400 and Varain Mercury 400 plus spectrometer at 400 MHz for ^1^H-NMR and at 100 MHz for ^13^C-NMR. The chemical shifts (δ) are assigned in comparison with residual solvent protons. The mass spectra were recorded by High Resolution ESI-QTOF Mass Spectrometer.

*6,8-dibromo-5,7-dihydroxyflavanone* (**TH011**): light brown powder (93% yield). ^1^H-NMR (CDCl_3_) δ 12.74 (s, 1H), 7.45 (m, 5H), 6.67 (bs, 1H), 5.58 (dd, *J* = 12.2, 3.5 Hz, 1H), 3.15 (dd, *J* = 17.3, 12.2 Hz, 1H), 3.00 (dd, *J* = 17.3, 3.5 Hz, 1H); ^13^C-NMR (CDCl_3_) δ 195.7, 159.3, 158.0, 157.4, 137.5, 129.1, 126.0, 103.9, 90.6, 89.0, 79.7, 42.6; HR-ESI-MS *m*/*z* calcd. for C_15_H_9_Br_2_O_4_Na [M + 2Na − H]^+^ 456.8663, found 456.8657.

*6,8-dibromo-5-hydroxy-7-methoxyflavanone* (**TH002**): yellow powder (96% yield). ^1^H-NMR (CDCl_3_) δ 12.61 (s, 1H), 7.48 (m, 5H), 5.61 (dd, *J* = 12.3, 3.4 Hz, 1H), 3.98 (s, 3H), 3.18 (dd, *J* = 17.3, 12.3 Hz, 1H), 3.05 (dd, *J* = 17.3, 3.4 Hz, 1H); ^13^C-NMR (CDCl_3_) δ 196.3, 162.1, 159.0, 157.7, 137.1, 128.8, 125.7, 105.9, 98.3, 96.6, 79.2, 60.7, 42.4; HR-ESI-MS *m*/*z* calcd. for C_16_H_12_Br_2_O_4_Na [M + Na]^+^ 448.90000, found 448.89938.

*6,8-diiodo-5-hydroxy-7-methoxyflavanone* (**TH012**): light green crystal (73% yield). ^1^H-NMR (acetone-*d_6_*) δ 13.02 (s, 1H), 7.47 (m, 5H), 5.85 (dd, *J* = 12.6, 3.2 Hz, 1H), 3.91 (s, 3H), 3.37 (dd, *J* = 17.3, 12.6 Hz, 1H), 3.14 (dd, *J* = 17.3, 3.2 Hz, 1H); ^13^C-NMR (acetone-*d*_6_) δ 198.2, 167.6, 164.0, 163.0, 139.2, 129.6, 127.1, 106.4, 95.61, 94.7, 80.5, 61.2, 42.6; HR-ESI-MS *m*/*z* calcd. for C_16_H_11_I_2_O_4_Na [M + 2Na − H]^+^ 566.8542, found 566.8525. 

*6-iodo-5-hydroxy-7-methoxyflavanone* (**TH018**): yellow oil (10% yield). ^1^H-NMR (acetone-*d*_6_) δ 12.95 (s, 1H), 7.53 (m, 5H), 6.33 (s, 1H), 5.68 (dd, *J* = 13.0, 3.1 Hz, 1H), 3.98 (s, 3H), 3.29 (dd, *J* = 17.4, 13.0 Hz, 1H), 2.92 (dd, *J* = 17.4, 3.1 Hz, 1H). ^13^C-NMR (acetone-*d*_6_) δ 195.3, 165.6, 164.8, 163.6, 162.2, 137.8, 129.0, 128.9, 126.1, 103.2, 93.2, 91.9, 79.4, 66.0, 42.6.

### 4.2. Antiviral Cell-Based Study 

#### 4.2.1. Cells and Viruses 

LLC/MK2 (ATCC^®^, Manassas, VA, USA CCL-7), HEK-293 (ATCC^®^ CRL-1573), HepG2 (ATCC^®^ HB-8065), THP-1 (ATCC^®^ TIB-202), and C6/36 (ATCC^®^ CRL-1660) cell lines were propagated and maintained as previously described ([9,11,37]. Huh-7 cells were maintained in DMEM (Gibco^®^, Langley, OK, USA) supplemented with 10% fetal bovine serum at 37 °C under 5% CO_2_. Reference DENV2 strains New Guinea C strain (NGC) and 16,681 were propagated in C6/36 cells, as described [9,11]. 

#### 4.2.2. Cytotoxic Concentration (CC_50_) Test 

LLC/MK2, THP-1, HEK-293, HepG2, or Huh-7 cells were seeded at 10^4^ cells per well of 96-well plate and incubated overnight. Compounds were prepared to 6–10 different concentrations in filter-sterilized dimethylsulfoxide (Merck^®^, Darmstadt, Germany) before addition to the cells. The plates were incubated for 48 h before the MTS reagent (Promega^®^, Madison, WI, USA) was added to cells according to the manufacturer’s protocol and incubated for 4 h before analysis by spectrophotometry at *A*_450nm_. Huh-7 cytotoxicity was assessed by the Alamar Blue assay (Invitrogen^®^, Waltham, MA, USA) and read by fluorometry at the excitation and emission wavelengths at 570 and 600 nm, respectively [38]. Each compound was tested in triplicate. Cytotoxic concentrations (CC_50_) were calculated while using non-linear regression analysis and the results were reported as means and standard deviation of three independent experiments.

#### 4.2.3. Effective Concentration (EC_50_) Test 

LLC/MK2 were seeded at 5 × 10^4^ cells per well of 24-well plate in growth medium and incubated overnight at 37 °C under 5% CO_2_. Cells were infected with DENV2 NGC at the multiplicity of infection (MOI) of 0.1 for 1 h with gentle rocking every 15 min. Cells were washed with PBS and incubated with MEM supplemented with 1% fetal bovine serum, 100 I.U./mL penicillin, and 100 μg/mL streptomycin. The compound was added to the virus-infected cells during and after infection. The cells were incubated for 72 h, unless otherwise indicated, at 37 °C under 5% CO_2_. Supernatants were collected and the viral infectivity was analyzed by 96-well plaque titration [39]. Data were plotted and the EC_50_ values were calculated by nonlinear regression analysis. Each concentration was tested in duplicate and the results were reported as the means and standard deviation of three independent experiments. The selectivity index was calculated from the ratio of CC_50_ and EC_50_. Moreover, additional EC50s of **TH002** and **TH011** were also taken from Huh-7 (8 × 10^4^ cells) and DENV2 16,681 (MOI of 1). Supernatants were collected after 72 h incubation for plaque titration EC_50_ values were calculated by nonlinear regression analysis. Data were presented by means and standard errors of three independent experiments.

#### 4.2.4. Attachment Inhibition Study 

Huh-7 were seeded at 8 × 10^4^ cells per well of 24-well plate in DMEM supplemented with 10% FBS and incubated overnight at 37 °C under 5% CO_2_. The cells were infected with the DENV2 16,681 at the MOI of 1 for 1 h at 37 °C. Compounds were diluted in DMSO and pre-incubated with the virus at 37 °C for 1 h, or added to the infected cells during or after the infection. The cells were washed with PBS and then incubated with DMEM supplemented with 2% fetal bovine serum for 72 h at 37 °C under 5% CO_2_. DMSO was used as a mock treatment. Supernatants were collected and the viral infectivity was analyzed by plaque titration. Three independent experiments were performed in order to verify the results. 

#### 4.2.5. Fusion Inhibition Study 

C6/36 were seeded at 1 × 10^5^ cells per well of 24-well plate MEM that was supplemented with 10% FBS and incubated overnight at 28 °C. Cells were infected with the virus at the multiplicity of infection (MOI) of 1 for 1 h at 28 °C. Compounds **TH002** and **TH011** were diluted in DMSO and added to the infected cells to the final concentration of 10 µM in 1% DMSO after DENV2 NGC (M.O.I. of 1) infection. Cells were incubated with MEM supplemented with 1% fetal bovine serum for 48 h before the addition of 0.5 M MES. Cells were incubated for an additional 24–48 h at 28 °C until the fused cells were visualized under light microscope. 

### 4.3. Animal Toxicity Study 

The animal care and use protocol identification number 016-2562 was approved by the Institutional Animal Care and Use Committee (CU-ACUP) of the Faculty of Medicine, Chulalongkorn University, Bangkok, Thailand, based on the National Institutes of Health, USA’s criteria for the use and treatment of laboratory animals. Male 8-week-old C57BL/6 mice purchased from the National Laboratory Animal Center, Nakhornpathom, Thailand were used in the experiments.

**TH011** and **TH002** were diluted with 10% DMSO in normal saline solution. The preparations were intravenously administered at the concentration of 10 mg/kg through a tail vein (*n* = 5/group). The 10% DMSO in normal saline solution was used as vehicle control. Blood were collected through tail vein nicking at day one, three, and eight after administration and the serum samples were kept at −80 °C until analysis. Renal function (serum creatinine; Scr) and liver function (serum alanine transaminase; ALT) were measured by colorimetric assays while using QuantiChromTM (DICT-500, BioAssay, Hayward, CA, USA) and EnzyChrom (EALT-100, BioAssay), respectively.

### 4.4. Computational Studies 

#### 4.4.1. Molecular Docking 

The crystal structures of all four interested DENV proteins were downloaded from the protein databank (PDB), including E protein (1OKE), NS2B/3 protease (2FOM), NS5 MTase (5EHI), and Pol domain (3VWS). The three-dimensional (3D) structures of all flavanones and reported inhibitors were constructed using GaussView 6 program and they were optimized at the B3LYP/6-31G* basis set [27] using Gaussian 16 software [40]. Each flavanone was docked with 100 independent runs into the four targets using AutoDock VinaXB [23] and compared with the known inhibitor in order to predict the binding pattern and affinity. The docking grid dimension; 20 × 20 × 20 = 8000 Å^3^ were set as the previously reported binding sites for each protein target [27]. The x, y, and z coordinates of each grid box are listed in Table S1 (in Supplementary Materials). Results were illustrated in the heatmap diagram. UCSF Chimera program [41] was used for visualization. The binding orientation of the compound with the highest binding affinity was selected as the initial structure for performing molecular dynamics (MD) simulation.

#### 4.4.2. Molecular Dynamics Simulation and FMO-RIMP2/C-PCM Calculation

The complex structures resulted from the molecular docking study were simulated for 300-ns under the periodic boundary condition (PBC) with the isothermal–isobaric (NPT) scheme while using the AMBER 16 program. The system preparation, minimization, and MD simulation at 300 K were set as previously described [42]. Protein was treated by AMBER ff14SB forcefield [43]. The partial charge of each ligand was prepared following the standard protocol [44], while the other parameters were taken from the general AMBER force field [45]. The 100 snapshots that were extracted from the last 100 ns were chosen to calculate the binding free energy using the MM/GBSA method [46]. From this set, the representative model of the potent flavanones taken from the clustering method based on pairwise best-fit root-mean-square deviations (RMSDs) of ligand and the residues within 5 Å around the ligand was further studied by the FMO-RIMP2/C-PCM method [47] while using the GAMESS program [48]. The intermolecular interaction energy (Pair interaction energy; PIEDA) is given by the sum of contributed energies; electrostatic ($E_{ES}$), charge exchange ($E_{EX}$), charge transfer ($E_{CT+MIX}$), dispersion ($E_{DI}$), and the solvation effect ($∆G_{Sol}^{C-PCM}$) [43,49]

## 5. Conclusions

This is the first report of newly synthesized dibromopinocembrin and dibromopinostrobin as potential leads of dengue inhibitors with good antiviral efficacies, mild toxicities in multiple cell lines, and animal model. The proposed molecular target was the viral methyltransferase with the G83 residue binding to ^6^Br atom of the compounds. 

## 6. Patents

This work is under preparation for Thailand patent submission. 

## Figures and Tables

**Table 1:** Efficacies and cytotoxicity of pinocembrin and pinostrobin derivatives.

Compounds	Abbreviation	DENV2 NGC, (16681) EC_50_ (µM)	LLC/MK2 CC_50_ (µM)	SI NGC, (16681) (CC_50_/EC_50_)
6,8-dibromopinocembrin	**TH011**	2.0640 ± 0.7537,	67.2082 ± 0.9731	32.5621
		(4.5936 ± 1.8520)		(10.5201)
pinocembrin	**TH022**	15.45 ^1^	>100 ^1^	>6.47
6,8-dibromopinostrobin	**TH002**	5.8567 ± 0.5074,	>100.0000	>17.0745,
		(3.1933 ± 1.0971)		(23.9342)
6,8-diiodopinostrobin	**TH012**	2.8000 ± 1.5544	>10.0000 ^2^	>3.5714
6-iodopinostrobin	**TH018**	4.1020 ± 1.8376	>25.0000 ^2^	>6.0945
pinostrobin	**TH019**	10.7567 ± 1.63	78.7844 ± 2.9193	7.3242

^1^ Srivarangkul et al. 2018 [11] ^2^ Insoluble from indicated concentrations.

**Table 2:** Cytotoxicities of selected pinocembrin (**TH011**) and pinostrobin (**TH002**) derivatives.

Cell lines	TH011 CC_50_ (µM)	TH002 CC_50_ (µM)
THP-1	>100.0000	51.7889 ± 2.2990
HEK-293	93.1907 ± 5.9901	>100.0000
HepG2	>100.0000	49.9188 ± 2.7029
Huh-7	48.3277 ± 4.1129	76.4457 ± 7.2027

## Supplementary Materials

The following are available online at. Table S1: The grid box parameters for molecular docking using AutoDock VinaXB, Figure S1: NMR spectra of TH011, Figure S2: NMR spectra of TH002, Figure S3: NMR spectra of TH012, Figure S4: NMR spectra of TH018, Figure S5: Fusion inhibition assay treated by TH002 and TH011, Figure S6: Solubility comparison of pinocembrin (TH022) and chrysin.
