# Supplementary material for: Dibromopinocembrin and Dibromopinostrobin Are Potential Anti-Dengue Leads with Mild Animal Toxicity
Source: Molecules. 2020 Sep 11;25(18):4154. doi: 10.3390/molecules25184154 (PMC7571160; doi:10.3390/molecules25184154)
Supplement: Supplementary file 1 [file molecules-25-04154-s001.pdf]

Supplementary Materials

Table S1 The grid box parameters for molecular docking using AutoDock VinaXB

Protein	PDB code	Binding site	X	Y	Z	Native inhibitor
E protein	1OKE	K site [33]	-14.0	78.6	43.6	FN5Y [27]
		Y site [27]	-19.5	60.5	59.5	
NS2B/3 protease	2FOM	Allosteric site [34]	-7.0	-9.7	9.0	Compound 9 [25]
NS5 MTase	5EHI	SAM site [28]	32.3	48.5	10.2	Sinefungin [28]
NS5 RdRp	3VWS	Active site [26]	20.0	60.1	16.0	NITD-107 [26]

Mass Spectrum List Report

Analysis Info

Analysis Name	D:\Data\Data Service\190401\T70_RC4_01_2444.d	Acquisition Date	4/1/2019 7:49:59 PM
Method	nv_pos_5min_profile_190214.m	Operator	CU.
Sample Name	T70	Instrument / Ser#	micrOTOF-Q II 10335
Comment			

Acquisition Parameter

Source Type	ESI	Ion Polarity	Positive	Set Nebulizer	3.0 Bar
Focus	Not active	Set Capillary	4000 V	Set Dry Heater	200 ℃
Scan Begin	100 m/z	Set End Plate Offset	-500 V	Set Dry Gas	8.0 l/min
Scan End	1500 m/z	Set Collision Cell RF	250.0 Vpp	Set Divert Valve	Waste

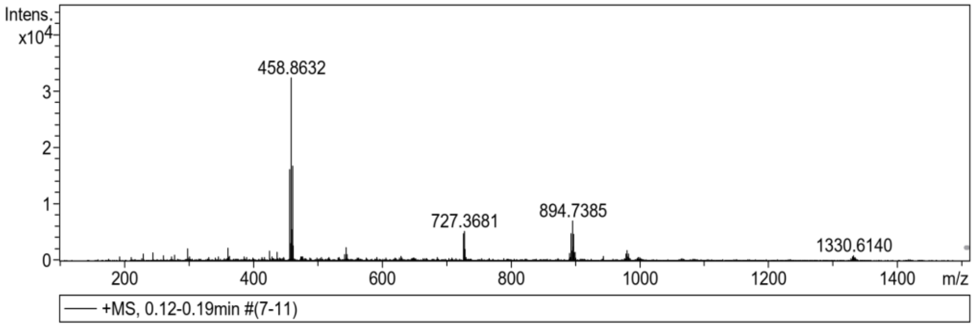

(a)

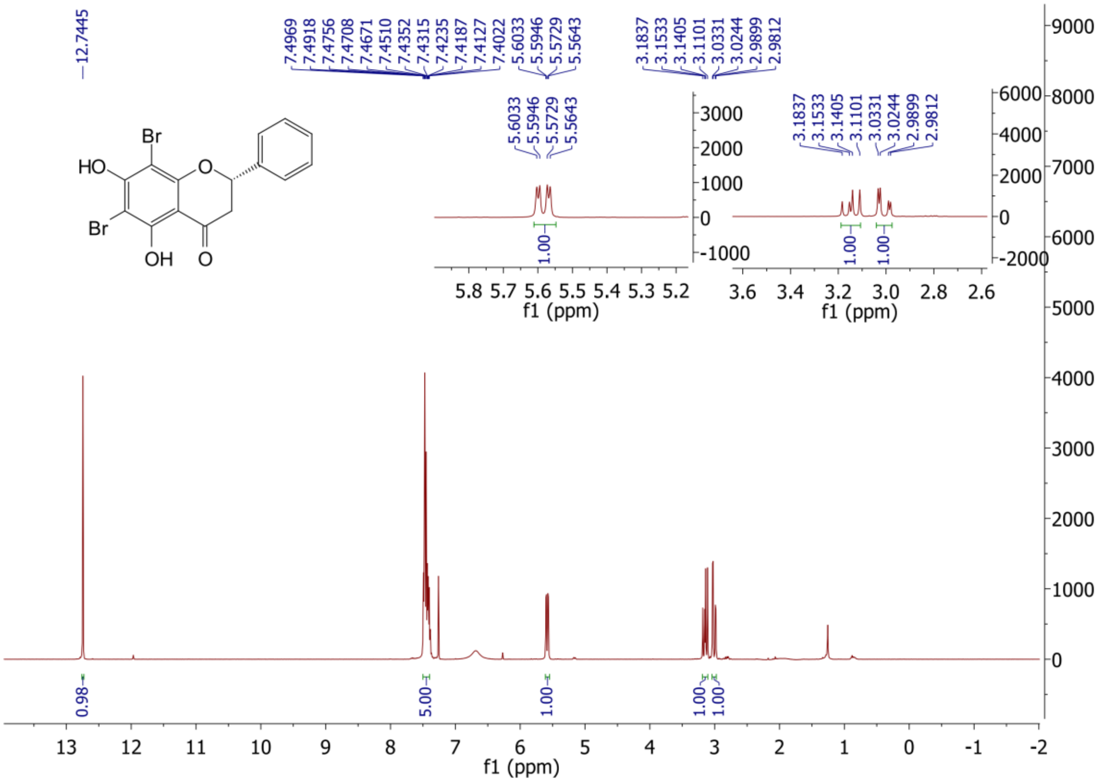

(b)

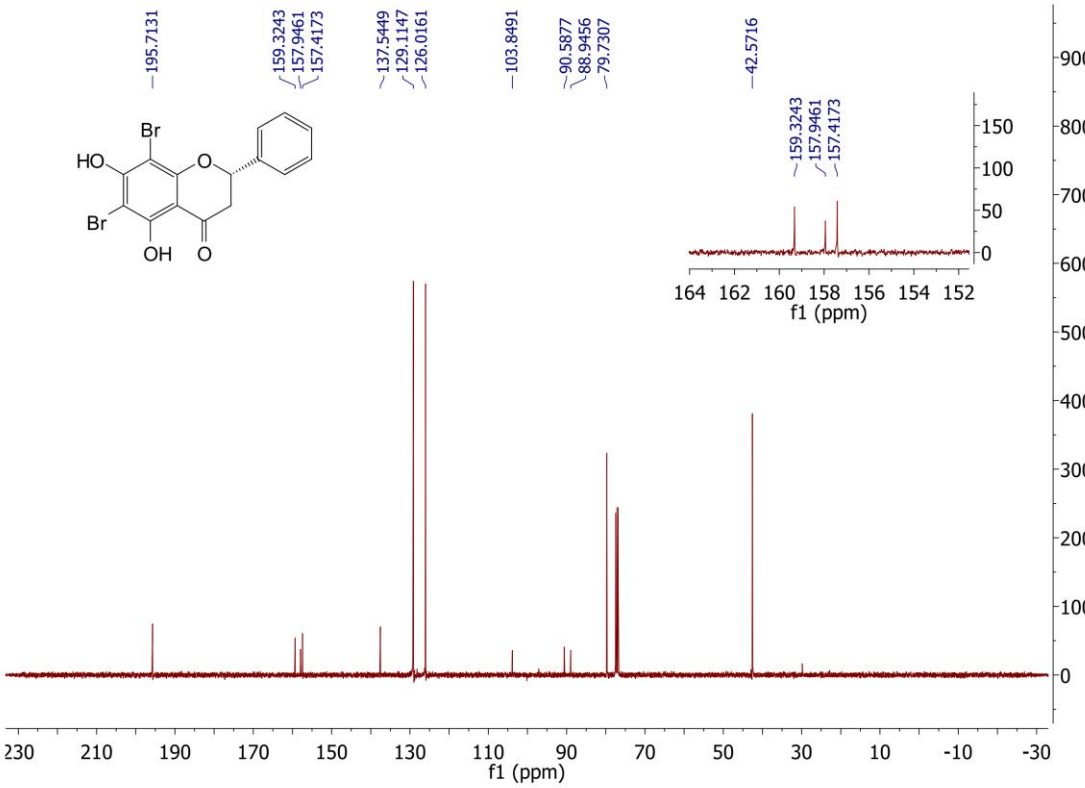

(c)

Figure S1. TH011 identification by (a) HR-ESI-MS. (b) ^{1}H NMR spectrum (Acetone-d_6, 400 MHz) (c)The ^{13}C NMR spectrum (Acetone-d_6, 100 MHz).

Mass Spectrum List Report

Analysis Info

Analysis Name	D:\Data\Data Service\180924_pos_T9.d	Acquisition Date	9/24/2018 1:20:00 PM
Method	NV_pos_0.3min_profile_1segment_lowNubulizerDrygas.m	Operator	CU.
Sample Name	180924_pos_T9	Instrument / Ser#	micrOTOF-Q II 10335
Comment			

Acquisition Parameter

Source Type	ESI	Ion Polarity	Positive	Set Nebulizer	0.4 Bar
Focus	Not active	Set Capillary	4000 V	Set Dry Heater	200 ℃
Scan Begin	50 m/z	Set End Plate Offset	-500 V	Set Dry Gas	4.0 l/min
Scan End	1500 m/z	Set Collision Cell RF	150.0 Vpp	Set Divert Valve	Waste

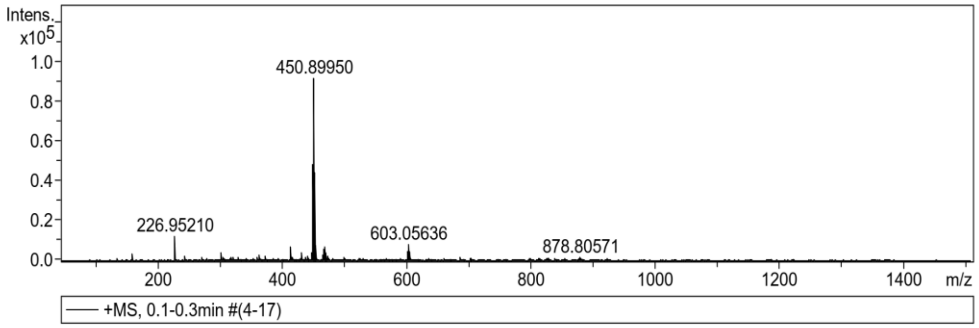

(a).

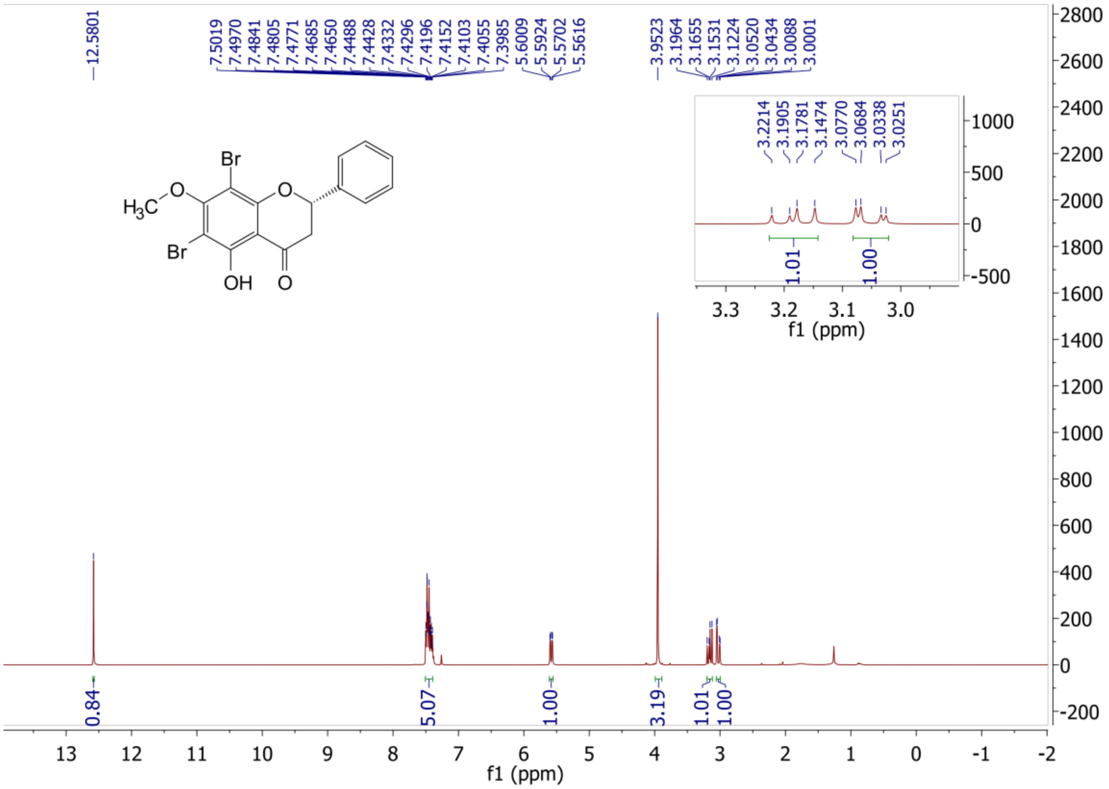

(b)

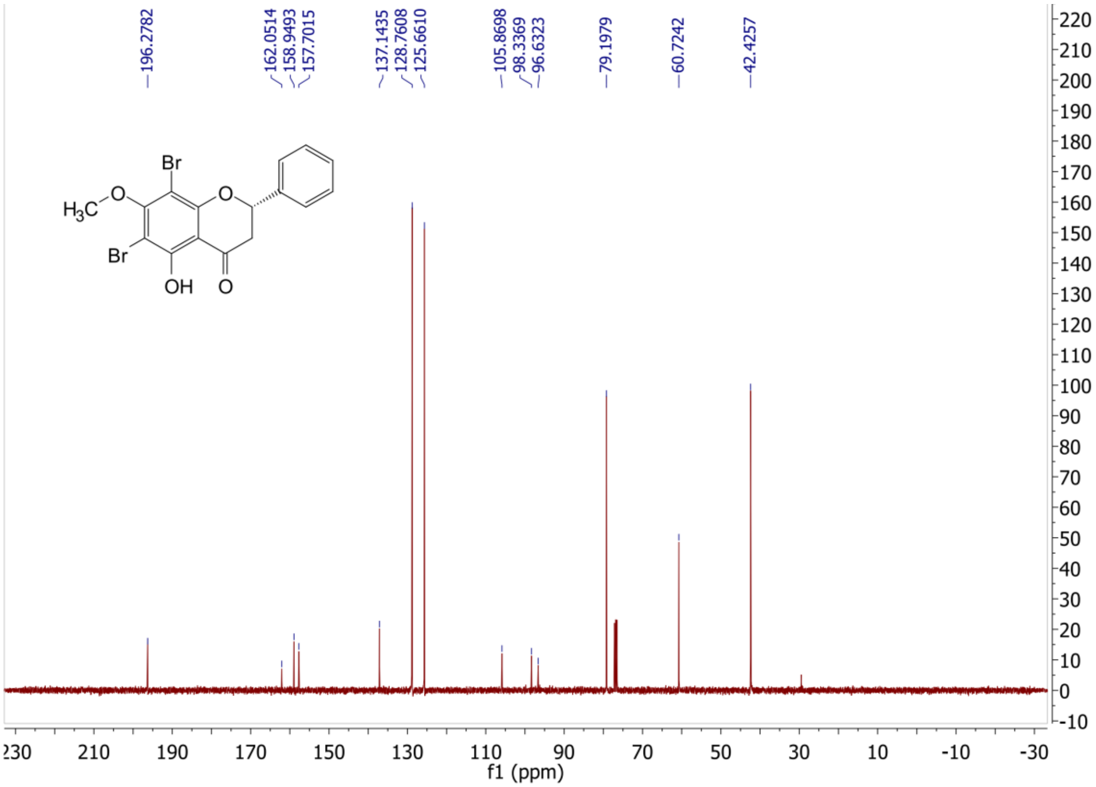

(c)

Figure S2. TH002 identification by (a) HR-ESI-MS. (b) ^{1}H NMR spectrum (Acetone-d6, 400 MHz) (c)The ^{13}C NMR spectrum (Acetone-d6, 100 MHz).

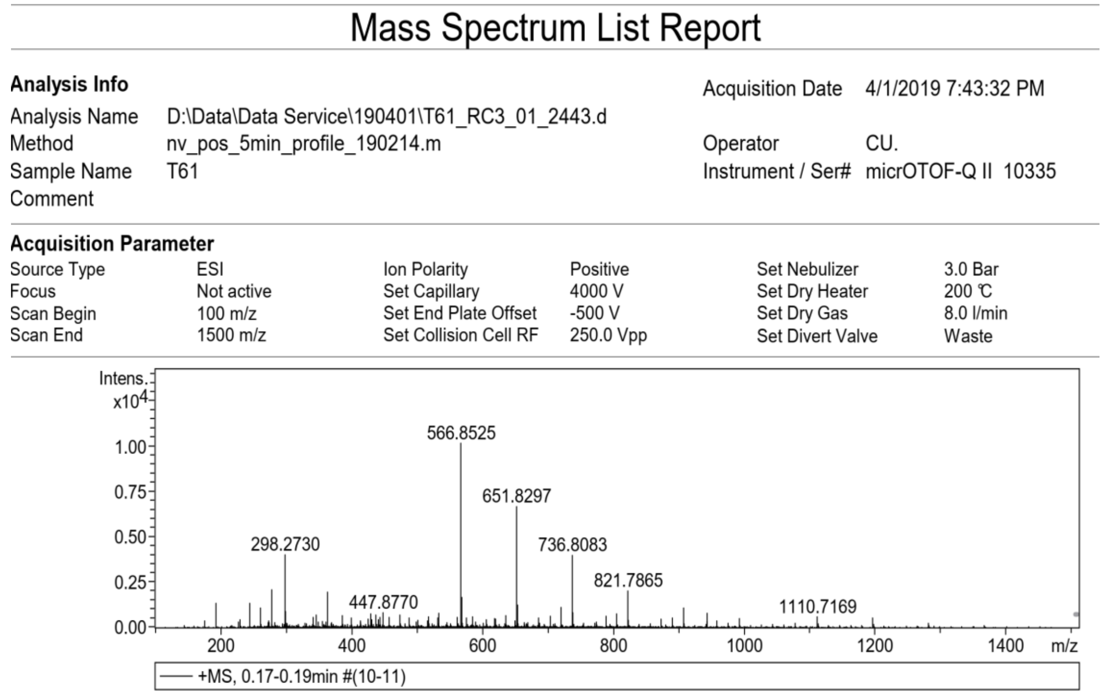
Mass Spectrum List Report
Analysis Info
Acquisition Date 4/1/2019 7:43:32 PM
Analysis Name D:\Data\Data Service\190401\T61_RC3_01_2443.d
Method nv_pos_5min_profile_190214.m
Operator CU.
Sample Name T61
Instrument / Ser# micrOTOF-Q II 10335
Comment
Acquisition Parameter
Source Type ESI
Ion Polarity Positive
Set Nebulizer 3.0 Bar
Focus Not active
Set Capillary 4000 V
Set Dry Heater 200 ℃
Scan Begin 100 m/z
Set End Plate Offset -500 V
Set Dry Gas 8.0 l/min
Scan End 1500 m/z
Set Collision Cell RF 250.0 Vpp
Set Divert Valve Waste
Intens. x10^4
566.8525
651.8297
298.2730
736.8083
821.7865
447.8770
1110.7169
m/z
+MS, 0.17-0.19min #(10-11)

(a)

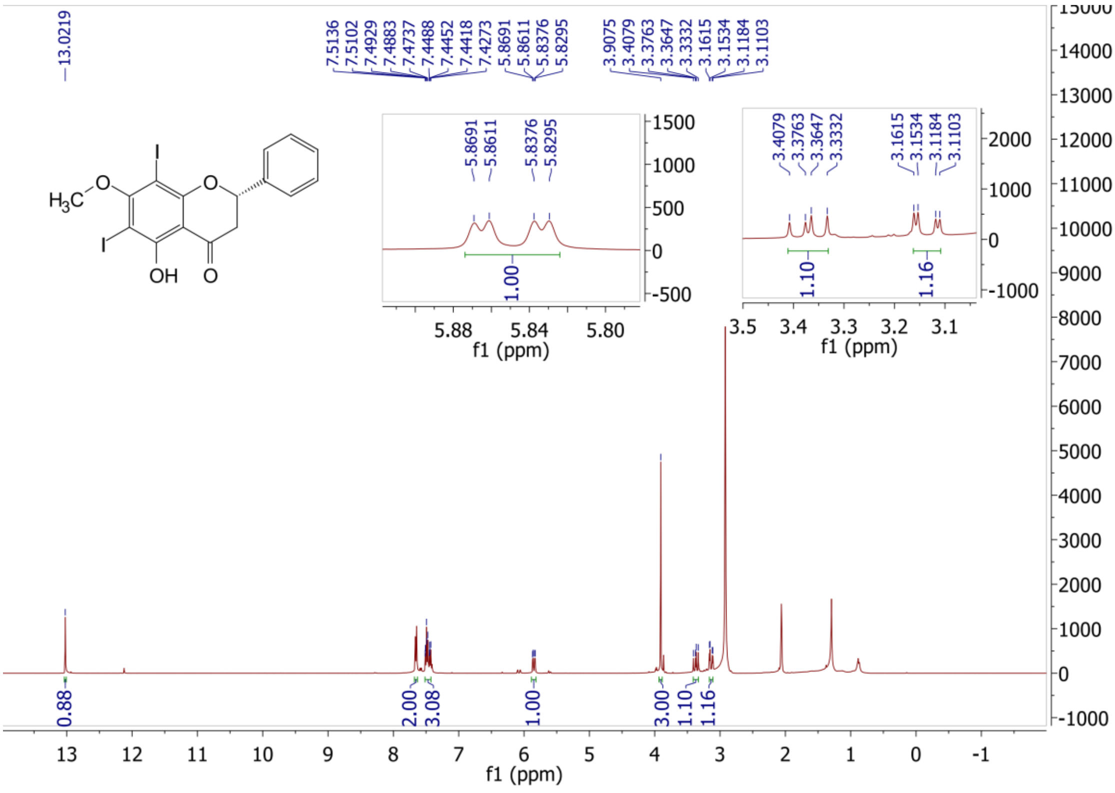
13.0219
7.5136
7.5102
7.4929
7.4883
7.4737
7.4488
7.4452
7.4418
7.4273
5.8691
5.8611
5.8376
5.8295
3.9075
3.4079
3.3763
3.3647
3.3332
3.1615
3.1534
3.1184
3.1103
H3C
O
I
OH
1.00
1.10
1.16
0.88
2.00
3.08
3.00
f1 (ppm)

(b).

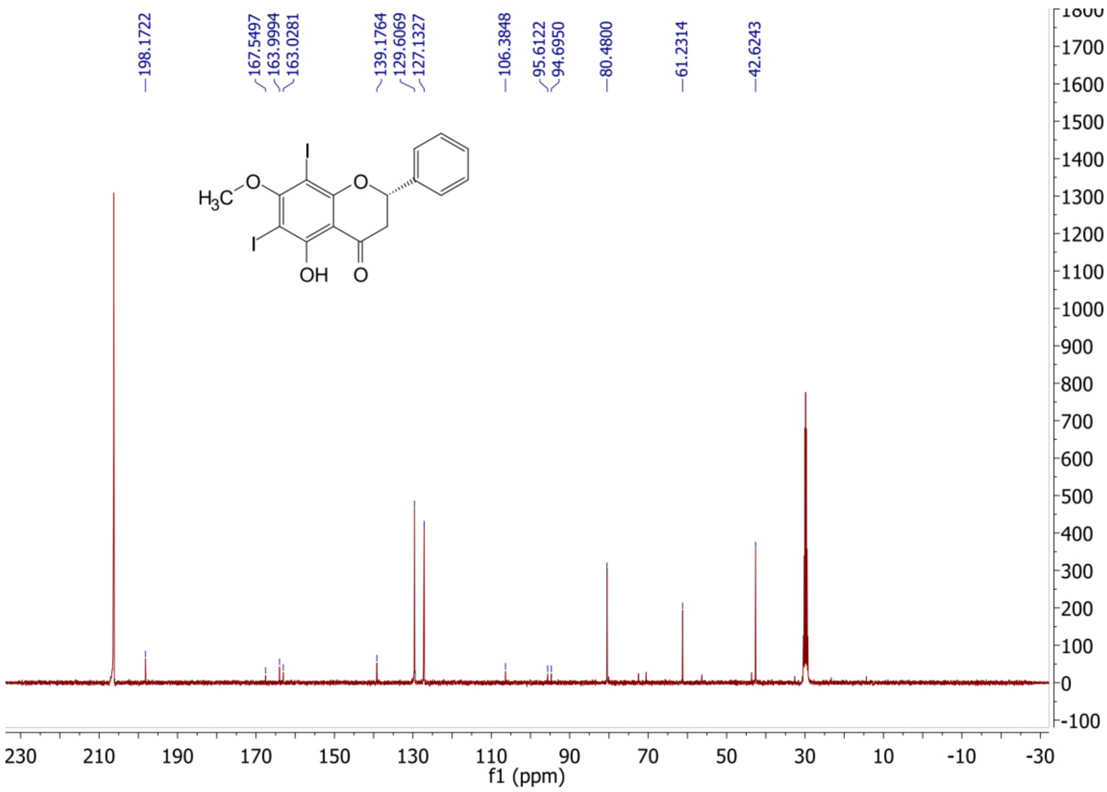

(c)

Figure S3. TH012 identification by (a) HR-ESI-MS. (b) ^{1}H NMR spectrum (Acetone-d_6, 400 MHz) (c)The ^{13}C NMR spectrum (Acetone-d_6, 100 MHz).

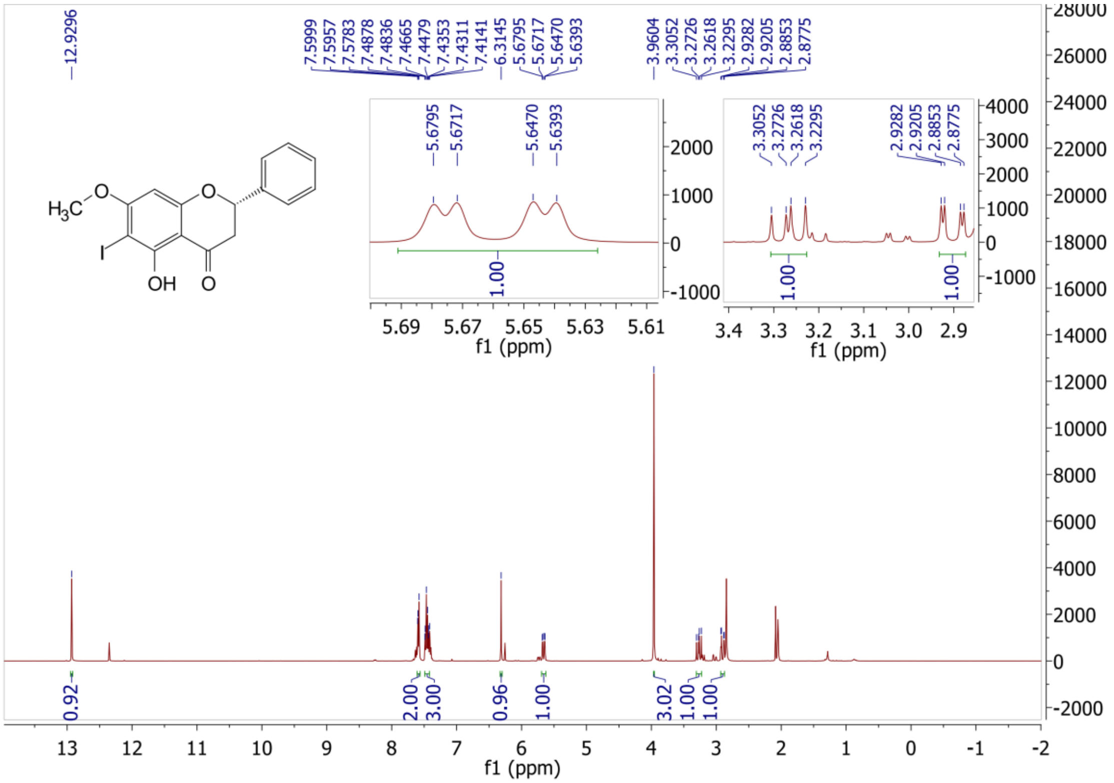

(a).

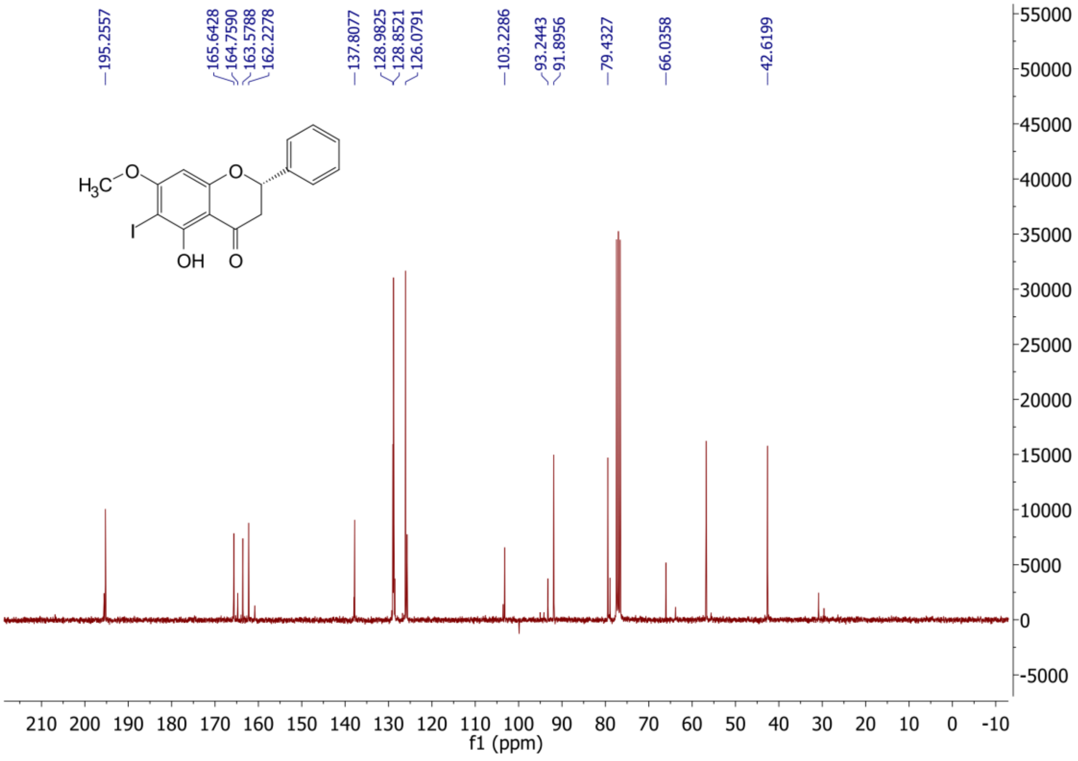

(b)

Figure S4. TH018 identification by (a) ^{1}H NMR spectrum (Acetone-d$_6$, 400 MHz) (b)The ^{13}C NMR spectrum (Acetone-d$_6$, 100 MHz).

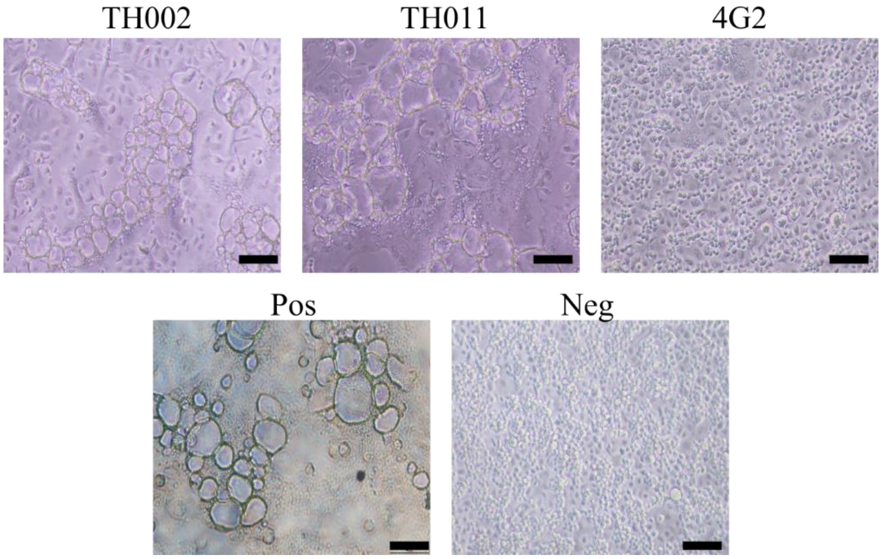

Figure S5. Fusion inhibition assay; TH002 and TH011 (10 μM) were incubated with the DENV2-infected cells for 2 days before addition of 0.5 M MES, pH 5.5. The 4G2 antibody was an inhibitor of DENV envelope fusion and was used as a control. Pos and Neg were abbreviated from positive (DENV) and negative (media-only) controls, respectively.

Pinocembrin (TH022)

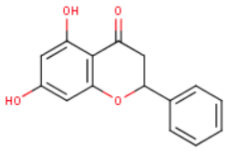

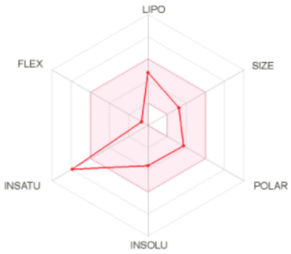

SMILES Oc1cc2OC(CC(=O)c2c(c1)O)c1ccccc1

Physicochemical Properties	
Formula	C15H12O4
Molecular weight	256.25 g/mol
Num. heavy atoms	19
Num. arom. heavy atoms	12
Fraction Csp3	0.13
Num. rotatable bonds	1
Num. H-bond acceptors	4
Num. H-bond donors	2
Molar Refractivity	69.55
TPSA	66.76 Å²

Lipophilicity	
Log $P_{o/w}$ (iLOGP)	2.11
Log $P_{o/w}$ (XLOGP3)	2.88
Log $P_{o/w}$ (WLOGP)	2.48
Log $P_{o/w}$ (MLOGP)	1.27
Log $P_{o/w}$ (SILICOS-IT)	2.55
Consensus Log $P_{o/w}$	2.26

Water Solubility	
Log *S* (ESOL)	-3.64
Solubility	5.81e-02 mg/ml ; 2.27e-04 mol/l
Class	Soluble
Log *S* (Ali)	-3.94
Solubility	2.93e-02 mg/ml ; 1.14e-04 mol/l
Class	Soluble
Log *S* (SILICOS-IT)	-4.00
Solubility	2.58e-02 mg/ml ; 1.01e-04 mol/l
Class	Soluble

Pharmacokinetics	
GI absorption	High
BBB permeant	Yes
P-gp substrate	No
CYP1A2 inhibitor	Yes
CYP2C19 inhibitor	Yes
CYP2C9 inhibitor	No
CYP2D6 inhibitor	No
CYP3A4 inhibitor	No
Log K_p (skin permeation)	-5.82 cm/s

Druglikeness	
Lipinski	Yes; 0 violation
Ghose	Yes
Veber	Yes
Egan	Yes
Muegge	Yes
Bioavailability Score	0.55

Medicinal Chemistry	
PAINS	0 alert
Brenk	0 alert
Leadlikeness	Yes
Synthetic accessibility	2.96

Chrysin

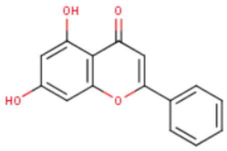

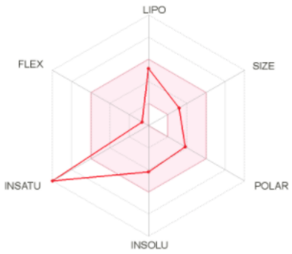

SMILES Oc1cc(O)c2c(c1)oc(cc2=O)c1ccccc1

Physicochemical Properties	
Formula	C15H10O4
Molecular weight	254.24 g/mol
Num. heavy atoms	19
Num. arom. heavy atoms	16
Fraction Csp3	0.00
Num. rotatable bonds	1
Num. H-bond acceptors	4
Num. H-bond donors	2
Molar Refractivity	71.97
TPSA	70.67 Å²

Lipophilicity	
Log $P_{o/w}$ (iLOGP)	2.27
Log $P_{o/w}$ (XLOGP3)	3.52
Log $P_{o/w}$ (WLOGP)	2.87
Log $P_{o/w}$ (MLOGP)	1.08
Log $P_{o/w}$ (SILICOS-IT)	3.02
Consensus Log $P_{o/w}$	2.55

Water Solubility	
Log *S* (ESOL)	-4.19
Solubility	1.64e-02 mg/ml ; 6.44e-05 mol/l
Class	Moderately soluble
Log *S* (Ali)	-4.69
Solubility	5.22e-03 mg/ml ; 2.05e-05 mol/l
Class	Moderately soluble
Log *S* (SILICOS-IT)	-4.98
Solubility	2.64e-03 mg/ml ; 1.04e-05 mol/l
Class	Moderately soluble

Pharmacokinetics	
GI absorption	High
BBB permeant	Yes
P-gp substrate	No
CYP1A2 inhibitor	Yes
CYP2C19 inhibitor	No
CYP2C9 inhibitor	No
CYP2D6 inhibitor	Yes
CYP3A4 inhibitor	Yes
Log K_p (skin permeation)	-5.35 cm/s

Druglikeness	
Lipinski	Yes; 0 violation
Ghose	Yes
Veber	Yes
Egan	Yes
Muegge	Yes
Bioavailability Score	0.55

Medicinal Chemistry	
PAINS	0 alert
Brenk	0 alert
Leadlikeness	No; 1 violation: XLOGP3>3.5
Synthetic accessibility	2.93

Figure S6. Solubility comparison of pinocembrin (TH022) and chrysin.
